# Exploratory study of tolerability and immunological effect of a short up-dosing immunotherapy phase with a standardised allergen extract derived from pollen of *Olea europaea*

**DOI:** 10.1186/s13601-015-0070-y

**Published:** 2015-07-24

**Authors:** Carmen Moreno, Blanca Sáenz De San Pedro, Carmen Millán, Carmen Panizo, Santiago Martín, Fernando Florido

**Affiliations:** Hospital Universitario Reina Sofía, Córdoba, Spain; Complejo Hospitalario de Jaén, Jaén, Spain; Hospital de Jerez de la Frontera, Cádiz, Spain; Servicio de Salud de Castilla La Mancha, Hospital Nuestra Señora del Prado, Talavera de la Reina, Toledo Spain; Medical Department, ALK-Abelló S.A., C/ Miguel Fleta, 19, Madrid, 28037 Spain; Hospital Universitario San Cecilio, Granada, Spain

**Keywords:** Allergen-specific immunotherapy, Allergen, Olive pollen allergy, Immune response, Skin reactivity, Tolerability, Seasonal allergic rhinitis

## Abstract

**Background:**

A new subcutaneous specific immunotherapy (SCIT) product adsorbed on aluminium hydroxide has been developed with a short and simplified up-dosing phase, containing a biologically standardized allergen pollen extract from *Olea europaea*.

**Objective:**

To assess the tolerability profile of the updosing phase and its immunological effect, in terms of specific IgG_4_ and IgE levels and immediate skin reactivity.

**Material and methods:**

The study was an exploratory, multi-centre, open-label, single-arm, phase II/III clinical trial. Adults with a clinical history of allergic rhinoconjunctivitis with/without asthma due to sensitization to olive pollen were selected. Five up-dosing doses (300, 600, 3000, 6000 and 15000SQ+) were administered at weekly intervals, followed by a maintenance dose (15000SQ+) after 2 weeks. Adverse events were collected during the 30 min observation period after injections, after a telephone contact 2 days after each visit, and after reviewing the subjects’ diary. IgG_4_ and IgE levels and immediate skin reactivity were evaluated at the beginning and at the end of the trial.

**Results:**

Ninety-three subjects were included in the trial (mean age, 35.7 ± 10.3 years; women, 66.7 %). A total of 95 adverse drug reactions, all mild in intensity and non-serious, were reported during the trial: 85 local in 34.4 % subjects, 9 systemic in 4.3 % subjects and one non-specific (grade 0). Within 6 weeks, significant changes in IgG_4_ and IgE levels and in immediate skin reactivity to *Olea europaea* were accomplished.

**Conclusion:**

This new SCIT derived from pollen of *Olea europaea* presented a good tolerability profile and induced significant immunological responses already after a 6 week treatment. However, the non-controlled design may limit the interpretation of these results.

**Trial registration:**

EudraCT no: 2011-004852-20; ClinicalTrials.gov Identifier: NCT01674595.

## Background

Allergic rhinoconjunctivitis and asthma are, frequently, concurrent disorders [1, 2], which has led to the notion that these two conditions are different aspects of the same disease [3, 4]. Results from the ONEAIR study, a prospective observational study conducted between 2004 and 2005 with 942 subjects from Spain, reinforced this idea, highlighting that almost 90 % of subjects suffering from asthma presented concomitant allergic rhinitis [5].

The ability of olive pollen (*Olea europaea*) to induce notorious symptoms of rhinitis and/or asthma in exposed populations through an IgE-mediated mechanism is amply documented in the Mediterranean areas [6–12]. A study conducted in 4000 allergic subjects from all over Spain showed that 47 % of subjects suffering from allergic rhinoconjunctivitis and 51 % of asthmatics were sensitized to *Olea europaea*, which emphasizes the importance of olive pollen allergen in Spain [13].

Available treatment options in respiratory allergic conditions include allergen avoidance, symptomatic treatment and etiologic treatment. Olive pollen allergen avoidance is hardly applicable since only a limited reduction in exposure can be achieved by modifying life habits. Symptomatic treatment offers short-term symptomatic relief, but does not offer long-term benefit, as the natural course of the disease is not affected. Etiologic treatment with allergen-specific immunotherapy (AIT) has proven to be the most effective treatment option for allergic diseases. AIT involves administering specific allergens to patients suffering from IgE-mediated allergic diseases in order to ameliorate symptoms, induce sustained and long-term immunological tolerance to the causative allergen, and potentially alter the natural course of the disease [14–16].

The subcutaneous route for the administration of AIT products has been proven to be clinically efficacious and well-tolerated in a number of studies [17–19]. The standard schedule for the subcutaneous immunotherapy (SCIT) starts with an induction period or up-dosing phase, involving increasing weekly doses over a number of consecutive weeks (12–14 weeks) until the maintenance dose is reached; however, there has been growing interest in reducing the initiation period to facilitate AIT compliance [20]. A new SCIT product containing allergen extract of *Olea europaea* (AVANZ® Olive) has been developed based on previous SCIT products.

Therefore, all the above considerations prompted us to assess the tolerability and the immunological effect of the up-dosing phase of this new SCIT containing allergen extract derived from olive pollen.

## Methods

### Study design

This was an open-label, single-arm, phase II/III multicenter clinical trial conducted at 10 sites in Spain, where olive pollen is an important cause of rhinitis and/or bronchial asthma. The study was approved by the applicable ethics committees and by the Spanish Drug Agency, and written informed consent was obtained from all subjects before their inclusion in the study. The trial was conducted in accordance with the Declaration of Helsinki [21] and in compliance with the Good Clinical Practice Guidelines [22]. The trial was registered on ClinicalTrials.gov under identification number NCT01674595.

### Study population

The study population comprised adult subjects aged 18 to 65 years with a clinical history of olive pollen-induced allergic rhinoconjunctivitis and/or asthma at least 1 year prior to trial entry, a positive skin prick test (SPT) response to *Olea europaea* (wheal diameter ≥ 3 mm), and a positive specific IgE against olive pollen (≥ IgE class 2; ≥ 0.70 KU/L) documented within the last 5 years. Subjects with forced expiratory volume in 1 s (FEV1) < 70 % of the predicted value at screening, uncontrolled or severe asthma, and a history of severe asthma exacerbation [23] were not included in the study. Subjects were also excluded if they had had an emergency room visit/admission because of asthma within the previous 12 months, a history of anaphylactic shock due to food, insect venom, exercise or drug, or severe and recurrent angioedema, or previous treatment with olive AIT within the previous 5 years or other concomitant AIT.

### Interventions

The trial was initiated after the olive pollen season of 2012. AVANZ® Olive injections were given by trained nurses and under supervision of expert allergists in Immunotherapy Units following national and international recommendations [24].

Subjects were administered 5 weekly subcutaneous up-dosing injections (300 SQ+, 600 SQ+, 3000 SQ+, 6000 SQ+ and 15,000 SQ+), followed by the maintenance injection (15,000 SQ) 2 weeks after, as part of the short course of SCIT (6 weeks). Two days after each visit, subjects were contacted by telephone to record any adverse event.

### Evaluations

Tolerability (primary endpoint) was assessed throughout the study as the incidence of adverse drug reactions (ADR) recorded during the 30 min waiting period after each injection, through phone calls 2 days after each injection and by reviewing the patients’ diaries issued to record any untoward experience. ADRs were defined as all noxious and unintended responses to any dose of the Investigational Medicinal Product (IMP) administered, and were classified as *immediate* (within 30 min after the injection) or *delayed* (>30 min after the injection). Likewise, ADRs were classified as *local* (LR, reactions occurring at the injection site), or *systemic* (SR, generalised signs/symptoms occurring away from the injection site). All LRs were recorded, regardless of size, including (diffuse) swelling, redness (erythema), pain, itching (pruritus) or injection site reaction (if two or more local symptoms occurred simultaneously); SRs were graded by the investigator according to the European Academy of Allergy and Clinical Immunology (EAACI) guidelines [24]. Additionally, all AEs where coded according to the Medical Dictionary for regulatory Activities (MedDRA).

At SCIT initiation (visit 1) and 6 weeks after the beginning of SCIT (visit 6), SPT to 5-fold concentrations of *Olea europaea* allergen extracts (12, 60 and 300 μg/ml Ole e 1) was performed and blood samples were taken for immunological assessments (secondary endpoint), including *Olea europaea*-specific IgG_4_ and IgE levels by ImmunoCAP (Phadia AB Uppsala). Changes in immediate skin response to *Olea europaea* were analyzed using the parallel line assay (PLA) [25], expressed as the cutaneous tolerance index (CTI), which indicates the difference in allergen concentration needed to elicit the same response.

### Statistical considerations

Statistical analyses were performed on the full analysis set of subjects, using the available data without imputation of missing values. The tolerability profile was determined using descriptive analyses. Changes in specific IgG_4_ and IgE levels between visit 1 and visit 6 were performed using Student’s *t*-test for paired samples. All statistical analyses were performed using the Statistical Package for the Social Sciences (SPSS) version 17.

## Results

**Fig. 1:**
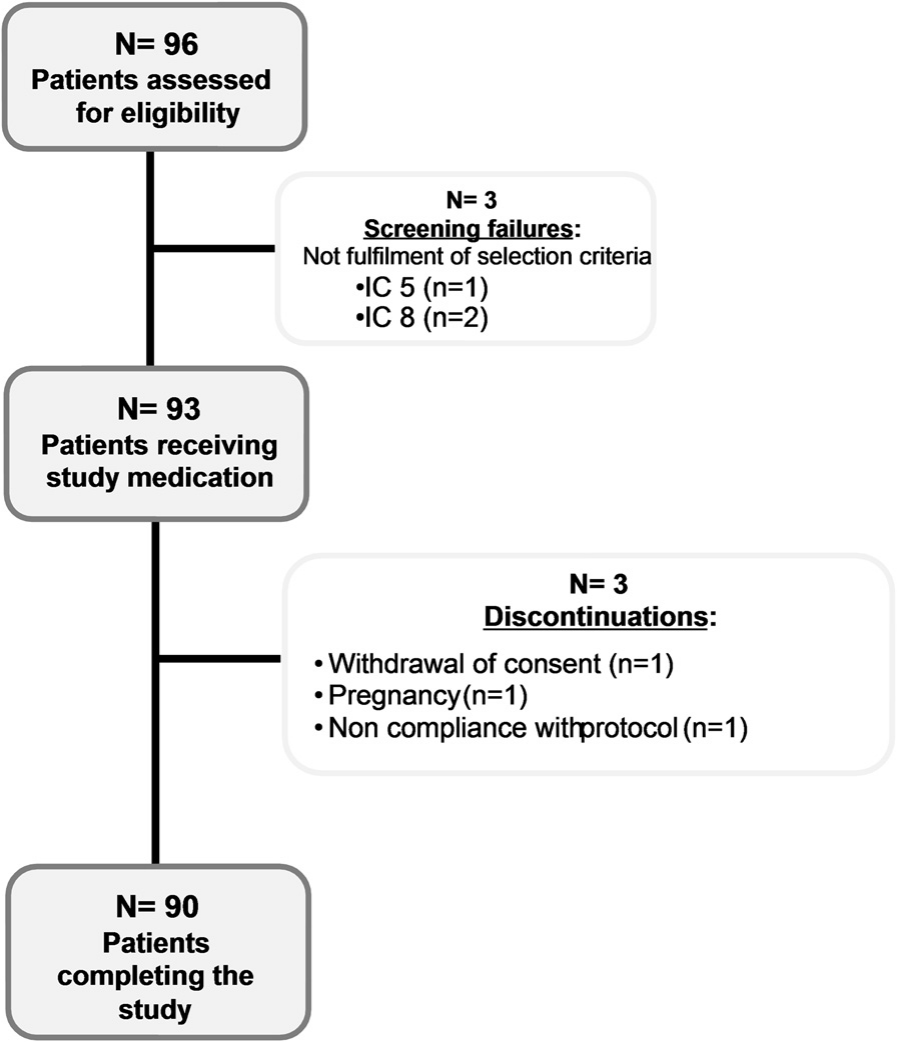
Study design flow chart

From September 2012 to April 2013, a total of 96 subjects were enrolled in the trial; as three subjects failed screening, 93 subjects were administered the IMP. Of these, three subjects discontinued before completing the study treatment (Fig. 1). The subjects’ baseline characteristics are presented in Table 1.

**Table 1:** Subjects’ baseline characteristics (*N* = 93)

Baseline characteristics	Value
Age (years), mean ± SD	35.7 ± 10.3
Women, n (%)	62 (66.7)
Ethnic origin, n (%):	
Caucasian	89 (95.7)
Hispanic	3 (3.2)
Arabian	1 (1.1)
Main concomitant illness, n (%):	
Asthma	64 (68.8)
Rhinoconjunctivitis	93 (100)
BMI (kg/m^2^), mean ± SD	25.9 ± 4.2
Vital signs, mean ± SD:	
Systolic blood pressure (mmHg)	120.2 ± 13.7
Diastolic blood pressure (mmHg)	73.7 ± 8.8
Heart rate (bpm)	74.2 ± 8.1
Smoking habits, n (%):	
Non-smoker	72 (77.4)
Smoker	14 (15.1)
Previous smoker	7 (7.5)
IgE *Olea europaea* CAP class, n (%):	
2	16 (17.2)
3	32 (34.4)
4	28 (30.1)
5	12 (12.9)
6	5 (5.4)

*BMI* body mass index, *bpm* beats per minute, *n* (%) number and percentage of subjects, *SD* standard deviation

### Tolerability

Thirty-four subjects (36.6 %) of IMP-exposed participants reported a total of 95 ADRs during the trial (Table 2). All ADRs were mild in intensity and non-serious, and all participants recovered fully. In one subject dose adjustment due to ADRs was done. A total of 85 local ADRs were reported by 32 (34.4 %) subjects without leading to changes in the administration schedule. Nine systemic ADRs were reported in 4 (4.3 %) subjects, all mild, non-serious, delayed and EAACI grade I. One subject experienced 4 SRs with different doses, two subjects two SRs each with the same dose and one subject only one SR. All dose concentrations elicited SRs and no risks were associated with a particular dose. SRs consisted in asthma symptoms and oticus and pharyngeal pruritus. Table 3 shows the nature of local and SRs by MedDRA System Organ Class (SOC) and Preferred Term (PT). One subject reported a non-specific (grade 0) ADR. No subjects required treatment with adrenaline and no safety concerns were raised for vital signs. IMP-unrelated AEs accounted for 84 events in 34 subjects.

**Table 2:** Summary of adverse drug reactions

	e	n (%)
IMP-related adverse events	95	34 (36.6)
Severity		
Mild	95	34 (36.6)
Moderate	0	0
Severe	0	0
Change in treatment schedule		
None	93	33 (35.5)
Temporarily interrupted	2	1 (1.1)
Discontinued	0	0
Prior to first intake	0	0
Classification according to EAACI guideline		
LR	85	32 (34.4)
SR	9	4 (4.3)
Grade 0/Nonspecific	1	1 (1.1)
Dose		
300 SQ+	16	13 (14.0)
600 SQ+	11	11 (11.8)
3,000 SQ+	24	22 (23.7)
6,000 SQ+	17	17 (18.3)
15,000 SQ+	26	15 (16.1)
Dose unknown	1	1 (1.1)

*ADR* adverse drug reaction, *e* number of events, *EAACI* European Academy of Allergy and Clinical Immunology, *n* (%) number and percentage of subjects, *LR* local reactions, *SQ+* standardized quality units, *SR* systemic reactions

**Table 3:** Nature of adverse drugs reactions

System organ class and preferred term	e	n (%)
Ear and labyrinth disorders		
Ear pruritus	1	1 (1.1)
General disorders and administration site conditions		
Asthenia*	1	1 (1.1)
Chest discomfort	1	1 (1.1)
Injection site erythema	1	1 (1.1)
Injection site pain	3	3 (3.2)
Injection site pruritus	25	12 (12.9)
Injection site reaction	52	22 (23.7)
Injection site swelling	4	3 (3.2)
Respiratory, thoracic and mediastinal disorders		
Asthma	4	1 (1.1)
Throat irritation	3	3 (3.2)

*e*, number of events; *n* (%), number and percentage of subjects

* EAACI Grade 0 (No symptoms or nonspecific symptoms of systemic reaction)

### Immunological effect

**Fig. 2:**
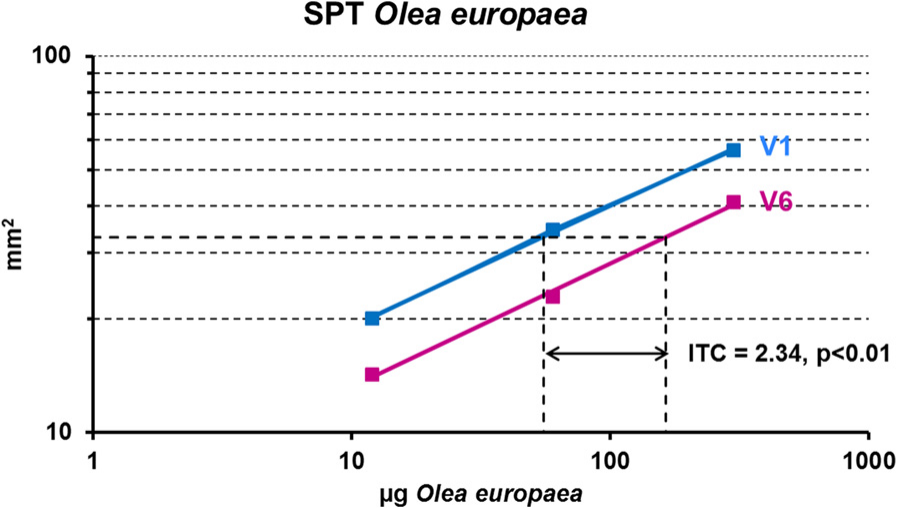
Changes in immediate skin reactivity by parallel line assay. Subjects were prick-tested with an *Olea europaea* pollen allergen extract containing 12, 60 and 300 μg/ml Ole e 1 before (V1) and after (V6) 6 week treatment. The reduction of skin reactivity was assessed by parallel line assay. CTI, Cutaneous Tolerance Index

A statistically significant increase of IgG_4_ and IgE levels to *Olea europaea* was observed from visit 1 to visit 6 (Table 4). Furthermore, within 6 weeks of treatment with *Olea europaea* SCIT, a significant reduction in immediate skin reactivity was observed with a CTI of 2.34 (95 % confidence interval (CI), 1.72 - 3.19). Changes in immediate skin reactivity over time are shown in Fig. 2.

**Table 4:** Levels of IgG_4_ and IgE to *Olea europaea*

	n	Visit 1 (mean ± SD)	Visit 6 (mean ± SD)	Effect size	*p*-value*
IgG_4_ (mg_A_/l)	90	0.34 ± 0.37	0.97 ± 1.13	0.64	<0.001
IgE (KU/l)	90	34.07 ± 36.09	47.50 ± 47.82	13.43	<0.001

*n* number of subjects with valid data in visit 1 and visit 6, *SD* standard deviation

*p-values correspond to paired Student’s *t*-test

## Discussion

Olive pollen is one of the most important causes of inhalant allergy in the Mediterranean countries [26] and is becoming the main disease-eliciting pollen in some provinces of Southern Spain (i.e. Córdoba, Jaén). The aim of this study was to determine the tolerability profile of the up-dosing phase of the new SCIT with *Olea europaea* allergen extract, measured as the incidence of ADRs. The trial was designed as non-controlled and as so, subjected to a certain degree of bias which may limit the interpretation of the results. However, regarding tolerability, the frequency of ADRs can be considered conservative, because all AEs with a temporary relation to the treatment with no other attributable cause have been related to the studied treatment. With regards to the immunological endpoints, the specificity of the immunological evaluations does not require a control group as it would be the case of a clinically-related endpoint.

The nature of the ADRs observed during treatment with this new SCIT was as expected. Over one-third of the study population suffered at least one ADR. All ADRs were mild in intensity, occurred at all dosing steps, most were related to the injection site and all participants achieved full recovery. Only one subject interrupted temporally the dosing schedule.

We have followed the current guidelines of the European Medicines Agency (EMA) on clinical development of AIT [27] and all AEs have been coded with MedDRA. Consequently, all local ADRs have been reported, independently of their size. These two factors, together with the way AEs were registered, proactively with a phone call 2 days after each injection and with a subject diary, may explain the apparently higher rate of ADRs in this trial. Although it is difficult to compare the safety profile between trials due to these differences in the safety reporting methodology, the pattern of the ADRs observed in this trial seems to be in line with previous experience and with what has been observed in previous clinical trials with other AIT products [28, 29].

A similar clinical trial led by Tabar AI [30], conducted with 102 subjects with the same SCIT formulation containing a house dust mite (*Dermatophagoides* mix)-derived allergen extract, showed similar results in terms of number and nature of the ADRs, with 4.9 % of the study population reporting mild, grade I SRs and a similar rate of LRs. In a randomised open controlled study of SCIT with a similar *Olea europaea* standardised allergen extract quantified in mass units performed with a conventional up-dosing schedule in a province of the Southern region of Spain (Jaén), where pollen counts may reach 7000 grains/m^3^, Gonzalez et al. [28] reported similar results with a rate of SRs of 8.7 %, with 3 mild and 1 moderate reactions.

One of the secondary endpoints of our study was to determine the immunological effect of this new SCIT formulation by measuring the specific levels of IgE and IgG_4_. Specific immunoglobulin levels are essential to establish the immune response of allergen immunotherapy, as the induction of IgG_4_ potentially blocks the proportion of IgE-facilitated antigen to T cells and eosinophils, resulting in a reduction of IgE production and the inflammatory response [29]. As might be expected, this new SCIT with olive pollen allergen extract induced immunological responses reflected as statistically significant increments of specific levels of IgE and IgG_4_ within 6 weeks of therapy. AIT often induces a short-term increase in IgE levels followed by a long-term decrease and a rapid increase in IgG_4,_ [19, 28, 31, 32] which can block the binding of IgE to allergens and B cells. The induction of T-regulatory cells (Treg) by AIT is central to the suppression of the allergic reaction: Treg produce IL-10 which is able to suppress IgE production by B cells and to induce IgG_4_ [33].

Several studies have evaluated the effect of SCIT on cutaneous reactivity to the causative allergen, showing continuous reduction in cutaneous response, which in some studies has been seen to correlate with clinical efficacy [34]. In our study, we achieved progressive reduction in immediate skin reactivity to the different concentrations of olive allergen extract, expressed as the CTI, i.e. the factor by which the extract concentration has to be multiplied to obtain the same response as in the beginning in a linear dose–response relationship. Recently, Tabar AI et al. [30] reported similar results, achieving a CTI of 1.44 (95 % CI, 1.04 - 1.98) after 6 weeks of immunotherapy. Likewise, clinical trials led by Martínez-Cocera [35] and Vidal [36] showed similar reductions in skin reactivity after a short course of AIT with allergen extract derived from *Phleum pratense* and house dust mites, respectively.

## Conclusion

The results of this phase II/III clinical trial show that immunotherapy with a biologically standardized allergen extract derived from *Olea europaea* in a 6 week schedule has a good tolerability profile and induces significant immunological responses in subjects suffering from allergic rhinoconjunctivitis and/or asthma due to sensitization to olive pollen.

## Abbreviations

ADR: Adverse drug reaction
AIT: Allergen-Specific Immunotherapy
CI: Confidence interval
CTI: Cutaneous tolerance index
EAACI: European Academy of Allergy and Clinical Immunology
EMA: European Medicines Agency
FEV1: Forced expiratory volume in 1 second
IMP: Investigational medicinal product
LR: Local reaction
MedDRA: Medical dictionary for regulatory activities
PLA: Parallel line assay
PT: Preferred term
SCIT: Subcutaneous immunotherapy
SOC: System organ class
SPSS: Statistical package for the social sciences
SPT: Skin prick test
SR: Systemic reaction
Treg: T-regulatory cells
